# Fatal Case of Glanders, with Remarks

**Published:** 1845-01

**Authors:** 


*Fatal Case of Glanders, with Remarks.*—A man, 55 years of age, was accidentally wounded in the cheek by one of the upper-jaw teeth of a glandered horse, while he was attempting to give the animal drink. The wound bled a good deal at the time; it was bathed first with salt and water, and then with flowers steeped in brandy. He remained quite well until the next afternoon, when he was seized with shivering and general *malaise.* He was hot and restless during this (the second) night and complained much of headache, pains in different parts of the body, more especially in the seat of the wound. The face became affected with erysipelas; the edges of the wound looked of a blueish cast, and were covered with phlyctenæ; there was exceeding prostration of strength and spirits; the breathing was hurried and oppressed, and the pulse very rapid and small. The pains in the joints, especially in the left hip, right knee, and lower part of the leg on this side, immediately above the ankle, were exceedingly severe.

For two or three days, the symptoms appeared to become mitigated under the treatment that was adopted; but on the seventh day we read that, in addition to the symptoms already enumerated, there was a sanious discharge from the right nostril, and petechial spots scattered over the surface of the body. The patient, who was every now and then delirious, frequently complained of intense pain in the hip, the integuments over which were swollen and œdematous. On the 13th day, the following is the report of the symptoms: dorsal decubitus, extreme prostration, profound stupor alternating with delirium, severe headache, eyelids œdematous and of a livid hue, the conjunctivæ deeply injected, incipient opacity of the corneæ; wound suppurating freely; the malar bone exposed; discharge of bloody matter from the nostrils; the nasal mucous membrane red, sprinkled over with sanguineous crusts, but without any visible ulcerated spots; lips sooty; tongue dry, yellowish and filthy; intolerable thirst; difficult deglutition; numerous brown papulæ, surrounded with red areolæ, over every part of the face, and confluent on the nose and lower eyelids; enormous "empatement," but without any sense of fluctuation of the left hip; distinct fluctuation immediately above the right ankle; a variolous-looking eruption on the abdomen and extremities, with phlyctenous and livid spots between the pustules.—The patient died next evening.

*Dissection.*—Passing over the description of the wound itself and of the cutaneous eruption, we may notice that the abscess above the right ankle was found to be situated under the fascia of the leg, and extended inwards into the substance of the muscles. The nasal mucous membrane was of a very dark colour, much softened in texture, and infiltrated with purulent matter; here and there were pustules and ecchymosed spots, especially on the surface of the spongy bones. The tonsils and the pillars of the fauces were bedewed with pus.—The epiglottis was œdematous, and speckled over, at one part, with minute sanguinolent spots.

The lining membrane of the larynx and trachea very highly congested, and dotted over with numerous whitish papulæ, like the incipient cutaneous pustules. Cellular texture underneath the sternum and pleura emphysematous; considerable serous effusion within the left pleura; lungs containing numerous subpleural purulent nuclei, which varied in size from that of a pea to that of a pigeon's egg, disseminated through their substance. The blood within the heart and large vessels was very black; but there were no coagula.

The reporter made some experiments on asses with the purulent discharge of this patient. Some of the pus from the nostrils and from the abscess near the right ankle was taken on the day preceding the patient's death, and inserted, by pretty deep scarifications into the flesh about the shoulders of a healthy and vigorous ass. The animal speedily exhibited all the symptoms of acute glanders, and died on the seventh day after the experiment.

The post-mortem appearances were in every respect such as are usually found after fatal cases of the idiopathic disease.

M. Landouzy quotes with approbation the remark of M. Bouley that "acute glanders is a highly contagious disease; contagious by the product of the nasal secretion; contagious by the expired air; contagious by the blood; and contagious by the tissues of the dead body. After the fever of incubation, when the virulent eruption takes place, the infected animal exsudes, so to speak, the morbific matter from every pore." The observations of MM. Rayer, Bresche, and others, have most satisfactorily shewed that the disease is transmissible to other animals besides those of the solipedous family; for example, to dogs, sheep and goats. A recent melancholy case at the Hôpital Necker clearly establishes the fact that it may be conveyed from one human subject to another; one of the *internes* at the hospital having died of the disease, caught by examining the body of an ostler who had fallen a sacrifice to it.

It appears that in three years from 1837 to 1840, no fewer than 27 persons have died in Paris of the glanders. M. Landouzy condemns the too common practice in the French metropolis, of feeding dogs and sheep with the flesh of horses that have died of the disease.

"As to any essential difference between the acute and chronic forms of glanders, we do not believe," says this gentleman, "that such exists; and the adoption of the contrary opinion has, on more than one occasion, led to a fatal security. We know of many indubitable cases of farcy communicated from horse to man, and *vice versâ*. If the identity of those two affections, and if the circumstance of their passing from the acute to the chronic state, and from the chronic to the acute, warrant certain scientific distinctions, they will not permit us, without great rashness, to establish any legal distinction."—*Ibid, from Gazette Medicale.*

